# Association of Hospice Payer With Concurrent Receipt of Hospice and Dialysis Among US Veterans With End-stage Kidney Disease: A Retrospective Analysis of a National Cohort

**DOI:** 10.1001/jamahealthforum.2022.3708

**Published:** 2022-10-21

**Authors:** Melissa W. Wachterman, Emily E. Corneau, Ann M. O’Hare, Nancy L. Keating, Vincent Mor

**Affiliations:** 1 Section of General Internal Medicine, Veterans Affairs Boston Health Care System, Boston, Massachusetts; 2 Division of General Internal Medicine, Brigham and Women’s Hospital, Boston, Massachusetts; 3 Department of Psychosocial Oncology and Palliative Care, Dana Farber Cancer Institute, Boston, Massachusetts; 4 Center for Healthcare Organization and Implementation Research, Veterans Affairs Boston Healthcare System, Boston, Massachusetts; 5 Long Term Services and Supports Center of Innovation, Veterans Affairs Providence Health Care System, Providence, Rhode Island; 6 Department of Medicine and Kidney Research Institute, University of Washington, Seattle; 7 Veterans Affairs Puget Sound Health Care System, Seattle, Washington; 8 Department of Health Care Policy, Harvard Medical School, Boston, Massachusetts; 9 Department of Health Services, Policy and Practice, Brown University School of Public Health, Providence, Rhode Island

## Abstract

**Importance:**

For many patients with end-stage kidney disease (ESKD), the Medicare Hospice Benefit precludes concurrent receipt of hospice and dialysis services, forcing patients to choose between continuing dialysis or enrolling in hospice. Whether the more liberal hospice eligibility criteria of the Veterans Health Administration’s (VA) are associated with improved access to concurrent dialysis and hospice care for patients with ESKD is not known.

**Objective:**

To examine the frequency of concurrent hospice and dialysis care among US veterans by hospice payer and examine the payer for concurrent dialysis.

**Design, Setting, and Participants:**

This was a retrospective cross-sectional study of all 70 577 VA enrollees in the US Renal Data System registry who initiated maintenance dialysis and died in 2007 to 2016. Data were analyzed from April 2021 to August 2022.

**Exposures:**

Hospice payer, either Medicare, VA inpatient hospice, or VA-financed community-based hospice (“VA community care”). Primary hospice diagnosis–ESKD vs non-ESKD.

**Main Outcomes and Measures:**

Concurrent receipt of hospice and dialysis services (“concurrent care”).

**Results:**

There were 18 420 (26%) eligible veterans with ESKD who received hospice services (mean [SD] age, 75.4 [10.0] years; 17 457 [94.8%] men; 2997 [16.3%] Black, 15 162 [82.3%] White, and 261 (1.4%) individuals of other races). Most of the sample (n = 16 465; 89%) received hospice services under Medicare and 5231 (28%) continued to receive dialysis after hospice initiation. The adjusted proportion of veterans receiving concurrent care was higher for those enrolled in VA inpatient hospice or VA community care hospice than it was for those enrolled in Medicare hospice (57% and 41% vs 24%, respectively; both *P* < .001). Regardless of hospice payer, the majority (87%) of the dialysis treatments after hospice initiation were financed by the VA, including for Medicare beneficiaries who had a hospice diagnosis other than ESKD. Median hospice length of stay was 43 days for veterans who received concurrent dialysis vs 4 days for those who did not.

**Conclusions and Relevance:**

In this retrospective cross-sectional study of US veterans with ESKD, a substantially higher proportion of veterans in VA-financed hospice received 1 or more dialysis treatments after hospice initiation than those enrolled in Medicare-financed hospice. Regardless of hospice payer, the VA financed most concurrent dialysis treatments. Hospice users who received concurrent dialysis care had substantially longer hospice lengths of stay than those who did not. These findings suggest that Medicare hospice policy may substantially restrict access to concurrent hospice and dialysis care among veterans with ESKD.

## Key Points

**Question:**

Does the frequency of receiving concurrent hospice and dialysis among veterans with end-stage kidney disease (ESKD) vary by hospice payer—Medicare, Veterans Health Administration (VA) inpatient hospice, or VA-financed community-based hospice?

**Findings:**

This retrospective cross-sectional study of a national cohort of 18 420 veterans with ESKD who received hospice found that patients receiving VA-financed hospice services were more likely to receive concurrent dialysis care than those receiving Medicare-financed hospice. Irrespective of hospice payer, the VA paid for the majority of concurrent dialysis treatments with which median hospice length of stay was 43 days vs 4 days without dialysis.

**Meaning:**

The findings of this retrospective cross-sectional study suggest that Medicare’s more restrictive hospice policy appears to limit access to concurrent dialysis and hospice care among veterans with ESKD and may be associated with a substantial reduction in length of hospice stay.

## Teaser

This retrospective cross-sectional analysis compares the use of concurrent dialysis and hospice by payer among US veterans with end-stage kidney disease and examines the association with length of stay.

## Introduction

When the Medicare Hospice Benefit was first established in 1982, it was explicitly intended to offer a less costly alternative to curative care.^1^ This intent was embodied in Medicare’s hospice eligibility criteria and payment policy. To be eligible for hospice services under Medicare, a physician must certify that a patient is expected to live for 6 months or less if the qualifying terminal illness (ie, the *primary hospice diagnosis*) follows its expected course. Under the Medicare Hospice Benefit, Medicare pays a hospice agency a per diem rate of approximately $200. The hospice agency is then expected to assume responsibility for all expenses related to the primary hospice diagnosis,^2,3^ while Medicare continues to cover the cost of care for unrelated conditions.

Unfortunately, Medicare’s approach to hospice eligibility and payment does not account for the complexities of serious illness, and it fails to recognize that some disease-directed therapies can have palliative benefits. The requirement to forfeit Medicare coverage for treatments related to one’s hospice diagnosis to receive hospice services—often referred to as Medicare’s “terrible choice”^4,5^—may be particularly challenging for patients with a terminal diagnosis such as end-stage kidney disease (ESKD). Because the cost of a single dialysis session far exceeds the Medicare per diem rate for hospice services,^6^ these patients are usually forced to choose between enrolling in hospice or continuing dialysis, a treatment without which most will survive only 1 to 2 weeks.^7,8^

In contrast to Medicare, the US Veterans Health Administration (VA)—as part of its Comprehensive End-of-Life Care Initiative launched in 2009—is committed to ensuring that seriously-ill patients who are veterans have access to hospice services, regardless of whether they are still receiving disease-modifying treatment.^9^ This commitment applies to all VA-enrolled veterans regardless of whether they receive VA- or Medicare-financed hospice services. Available data on concurrent receipt of hospice and chemotherapy or radiation therapy among veterans with advanced cancer suggest that concurrent care may improve the quality and outcomes of end-of-life care.^10,11^ To our knowledge, no study has previously examined use of concurrent dialysis and hospice services among veterans with ESKD.

Because most patients with ESKD are eligible for Medicare at any age, many VA-enrolled veterans have the option to receive hospice services under either their VA or Medicare benefits. In the context of the VA’s more liberal approach to hospice eligibility and payment, comparative patterns of concurrent care among veterans with ESKD receiving hospice services under Medicare vs VA could be informative. This study compared rates of concurrent dialysis and hospice use among a cohort of VA-enrolled veterans receiving maintenance dialysis for ESKD and examined which hospice payers financed dialysis treatments after hospice initiation.

## Methods

This study followed the Strengthening the Reporting of Observational Studies in Epidemiology (STROBE) reporting guideline for cross-sectional studies.^12^ This study was approved by the institutional review board of the Providence (Rhode Island) VA medical center and all linked Centers for Medicare & Medicaid Services data were obtained via a data use agreement with the VA Information Resource Center.

### Study Design, Data Sources, and Cohort

We conducted a retrospective cross-sectional study among a decedent cohort of VA-enrolled veterans with ESKD who initiated maintenance dialysis and died in 2007 through 2016. All participants were veterans whose records appeared in the US Renal Data System (USRDS), a comprehensive national registry of patients treated with maintenance dialysis and/or kidney transplant. Additional data sources included VA enrollment files, and outpatient encounters, claims for VA-financed non-VA community care, the VA Vital Status File, Medicare enrollment files, and Medicare inpatient, outpatient, carrier, and hospice claims.

**Figure 1.**
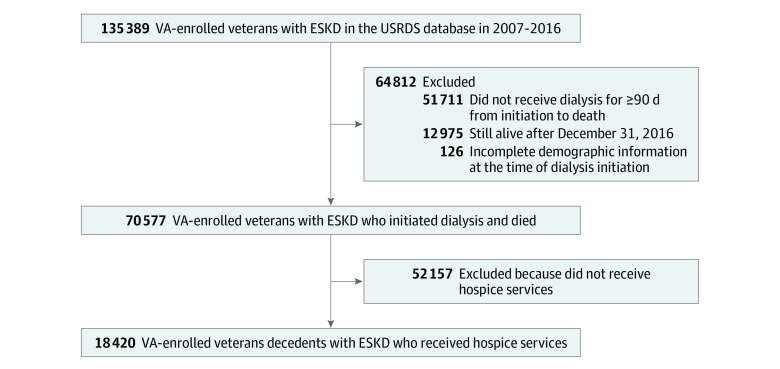
Study Participant Flow Diagram Abbreviations: ESKD, end-stage kidney disease; USRDS, US Renal Data System registry; VA, Veterans Health Administration.

As shown in Figure 1, 135 389 VA-enrolled veterans appeared in the USRDS registry during the observation period. Veterans were excluded if they died within 90 days of dialysis initiation (n = 51 711), were still alive after the follow-up period that ended on December 31, 2016 (n = 12 975), or their record were missing demographic information at dialysis initiation (n = 126). This yielded an analytic cohort of 70 577 veteran decedents with ESKD.

### Primary Outcomes and Covariates

Hospice services among all VA-enrolled veterans who appeared in the USRDS and died during the observation period were described; we then determined the proportion who received dialysis services after hospice initiation (concurrent care), by hospice payer. Veterans can receive hospice at a VA facility financed by the VA, or in the community, financed either by the VA (VA community care) or by Medicare. Because so few patients in the study cohort received hospice from more than 1 payer source (n = 351; 1.9%), patients were assigned to the payer that financed their first day of hospice services. Concurrent care was defined as having at least 1 dialysis treatment after hospice initiation. We calculated the duration of concurrent care as the number of days between hospice initiation and the last day of dialysis treatment. Hospice length of stay (LOS) was defined as the number of days between hospice initiation and death.

Demographic covariates included age, sex, and race (Black, White, or other); other race included Asian, Native Hawaiian or other Pacific Islander, American Indian or Alaska Native. Ethnicity (eg, Hispanic) was not considered. Race was ascertained using the patient files from the VA Corporate Data Warehouse and may have been recorded by self-report, proxy, or observer. Additional covariates included residence (rural/urban), defined by the Rural-Urban Commuting Areas system, and proximity to a VA facility, defined as the distance in miles from a patient’s residence to the nearest VAMC. Because dialysis payment source before hospice initiation might influence subsequent access to dialysis treatment (ie, concurrent dialysis), we examined rates of concurrent care by dominant dialysis payer, defined as the payer that financed the greatest number of dialysis treatments, categorized as either Medicare-financed dialysis (Medicare), dialysis at a VA facility (VA), or VA-financed dialysis in a non-VA facility in the community (VA community care). For patients who received concurrent care, we examined the primary dialysis payer *after hospice initiation* (concurrent dialysis payer), using the same categories.

### Statistical Analyses

First, we calculated the percentage of veteran decedents with ESKD who received hospice services, and, among hospice users, we described patient characteristics by hospice payer. We present unadjusted percentages of hospice users who received concurrent care by hospice payer, both overall and stratified by predominant dialysis payer. Also, we presented median hospice LOS, overall, by hospice payer, and by receipt of concurrent hospice and dialysis services. We used multivariable logistic regression models to compare adjusted proportions of hospice users who received concurrent care by hospice payer, both overall and stratified by predominant dialysis payer, after adjusting for age, race, sex, residence (rural/urban), and proximity to a VA facility. We also adjusted for fixed effects of Veterans Integrated Service Networks. Among patients who received concurrent care, we examined primary payment source for dialysis treatments *after hospice initiation* (“concurrent dialysis”), both overall and after stratifying by hospice payer.

### Subgroup Analyses Among Medicare-Financed Hospice Users

Because eligibility for concurrent dialysis under Medicare is contingent on the primary hospice diagnosis, we examined the percentage of Medicare-financed hospice users who had a primary hospice diagnosis of ESKD vs a non-ESKD condition, overall and after stratification by concurrent care use. Primary hospice diagnosis was based on the principal diagnosis code listed on the first hospice claim. Because primary hospice diagnosis was missing for 43.6% of VA-financed hospice users, we focused the analyses on Medicare-financed hospice users.

We also examined the unadjusted and adjusted percentages of hospice users who received concurrent care and hospice LOS after stratification by setting of Medicare hospice services to support comparisons with patients who received VA inpatient hospice. Setting of hospice care was defined using the facility type code from the first Medicare hospice claim, which was dichotomized as either home hospice or inpatient hospice.

All data analyses were performed from April 2021 to August 2022 using SAS Enterprise Guide, version 7.1 (SAS Institute), and Stata, version 15.0 (StataCorp, LLC). Statistical tests were 2-tailed and *P* values < .05 were considered statistically significant.

## Results

### Sample Characteristics

Of the analytic cohort, 18 420 (26.1%) veteran decedents with ESKD used hospice services prior to death (mean [SD] age, 75.4 [10.0] years; 17 457 [94.8%] men; 2997 [16.3%] Black, 15 162 [82.3%] White, and 261 [1.4%] individuals of other races; (Figure 1). Most of the hospice users (89.4%; n = 16 465 patients) received Medicare-financed hospice services; 2.0% (366) received VA community care hospice; and 8.6% (1589) received VA inpatient hospice (Table 1). Medicare-financed hospice users were older than VA-financed hospice users (mean age, 76.2 years vs 67.7 and 69.3 years for VA community care and VA inpatient, respectively; *P* < .001) and more were White individuals (83.7% vs 76.0% and 69.1%, respectively; *P* < .001; Table 1).

**Table 1.** Characteristics of VA-Enrolled Hospice Users Who Had Received Maintenance Dialysis, by Hospice Payer

Characteristics	Hospice payer, No. (%)				*P* value
	Total study population	Medicare	VA community care	VA inpatient	
No. (%)	18 420	16 465 (89.4)	366 (2.0)	1589 (8.6)	NA
**Demographic information**					
Mean age (SD) at death, y	75.4 (10.0)	76.2 (9.7)	67.7 (9.7)	69.3 (10.6)	<.001
Race					
Black	2997 (16.3)	2451 (14.9)	85 (23.2)	461 (29.0)	<.001
White	15 162 (82.3)	13 786 (83.7)	278 (76.0)	1098 (69.1)	
Other^a^	261 (1.4)	228 (1.4)	3 (0.8)	30 (1.9)	
Male sex	17 457 (94.8)	15 541 (94.4)	355 (97.0)	1561 (98.2)	<.001
Rural residence	2045 (11.1)	1891 (11.5)	42 (11.5)	208 (13.1)	.16
Nearest VAMC, mean (SD), m	39.4 (38.9)	34.5 (34.9)	42.6 (38.3)	31.3 (37.8)	<.001
**Dialysis payer** ^b^					
Medicare	14 487 (78.6)	14 055 (85.4)	80 (21.9)	352 (22.2)	<.001
VA community care	3007 (16.3)	2003 (12.2)	219 (59.8)	785 (49.4)	
VA	926 (5.0)	407 (2.5)	67 (18.3)	452 (28.4)	

Abbreviations: NA, not applicable; VA, US Veterans Health Administration; VAMC, VA Medical Center.

^a^ Included individuals who identify as Asian, Native Hawaiian or other Pacific Islander, and American Indian or Alaska Native.

^b^ Based on the payer that financed most of the veteran’s dialysis treatments.

### Hospice Payer and Concurrent Care

**Figure 2.**
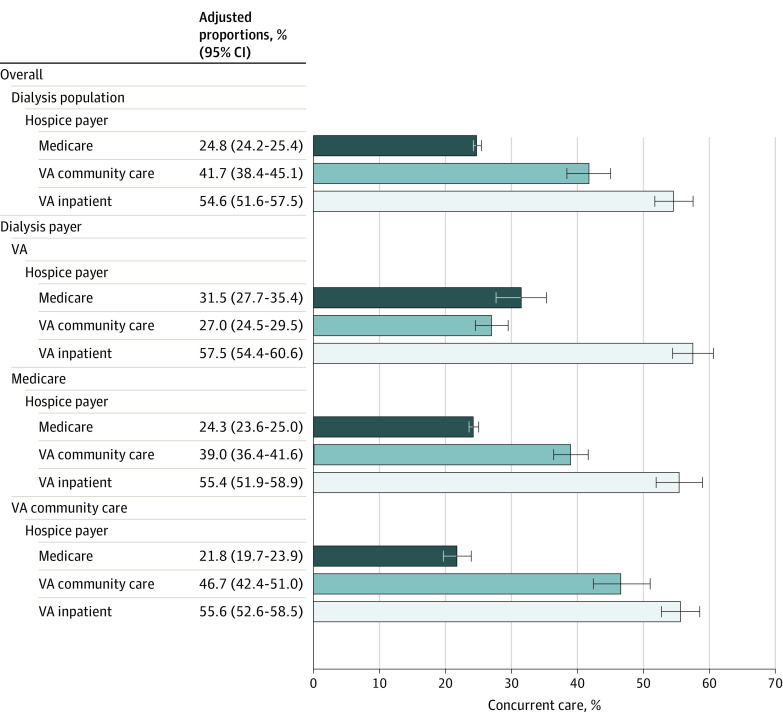
Adjusted^a^ Proportions (95% CIs) of VA-Enrolled Hospice Users Receiving Concurrent Care by Hospice Payer, Overall and Stratified by Predominant Dialysis Payer ^a^Adjusted for age, sex, race, rural, and distance to VA Medical Center with Veterans Integrated Services Networks fixed effects. Black bars denote 95% CIs.

Among the cohort of hospice users, 28.4% continued to receive dialysis after hospice initiation (Table 2). The adjusted proportion of hospice users who received concurrent care was lower for those receiving Medicare-financed hospice (24.8%; 95% CI, 24.2%-25.4%) than for those receiving VA-financed hospice, either VA community care (41.7%; 95% CI, 38.4%-45.1%) or VA inpatient hospice (54.6%; 95% CI, 51.6%-57.5%; *P* < .001 for both comparisons; Figure 2 and Table 2). Results were similar after stratification by predominant dialysis payer, with the exception that the adjusted proportion of Medicare-financed hospice enrollees receiving concurrent care was higher for veterans who received VA facility-based dialysis (40.4%; 95% CI, 34.9%-45.8%) than for those who received either VA community care dialysis (23.1%; 95% CI, 20.9%-25.2%) or Medicare dialysis (24.3%; 95% CI, 23.6%-25.1%; *P* < .001 for both comparisons; Figure 2). Unadjusted results were very similar (Table 2 and the eFigure in the Supplement).

**Table 2.** Concurrent Care (Dialysis and Hospice) Utilization and Length of Stay, by Hospice Payer

Variables	Total hospice sample	Hospice payer, No. %		
		Medicare	VA community care	VA inpatient
No. (%)	18 420	16 465 (89.4)	366 (2.0)	1589 (8.6)
**Concurrent care**				
Unadjusted, No. (%)	28.4 (5231)	24.6 (4050)	58.7 (215)	60.8 (966)
Adjusted,^a^ % (95% CI)	NA	24.8 (24.2-25.4)	41.7 (38.4-45.1)^b^	54.6 (51.6-57.5)^b^
**Length of stay, median (IQR), d**				
Overall	5 (17)	5 (15)	16 (72)	6 (62)
Hospice only	4 (6)	4 (6)	5 (12)	1 (9)
Concurrent care	43 (151)	47 (147)	48 (161)	17 (161)

Abbreviation: VA, Veterans Health Administration.

^a^ Adjusted for age, sex, race, residence (rural/urban), and proximity to a VA facility.

^b^ *P* < .001 for comparison vs Medicare-financed hospice.

**Figure 3.**
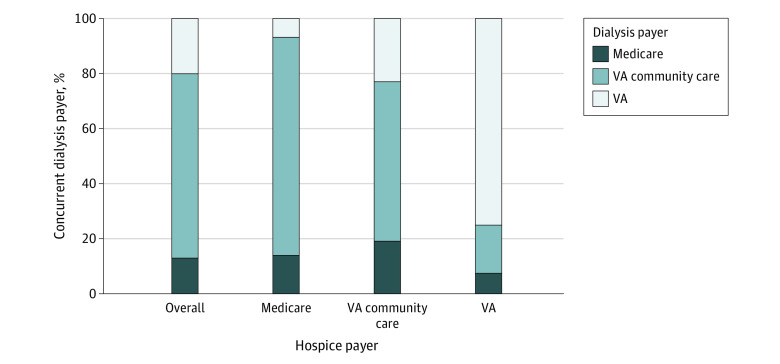
Dialysis Payer Among Hospice Users Receiving Concurrent Care, Overall and by Hospice Payer

Among the subset of veterans who received concurrent care, 87% of dialysis treatments provided *after hospice initiation* were financed by the VA regardless of hospice payer. The VA paid for the vast majority of concurrent dialysis treatments among those receiving Medicare-financed hospice services (Figure 3).

### Hospice Length of Stay and Concurrent Care

As shown in Table 2, the median hospice length of stay (IQR) for hospice enrollees was 5 (17) days overall—5 (15) days for Medicare-financed, 16 (72) days for VA community care, and 6 (62) days for VA inpatient. The median (IQR) hospice LOS was 43 (151) days for those who received concurrent care and 4 (6) days for those who did not. Among hospice enrollees who received concurrent care, median (IQR) hospice LOS was similar for those who received Medicare vs VA community care-financed hospice (47 [147] days and 48 [161] days, respectively) and shorter for those enrolled in VA inpatient hospice (17 [161] days). Among hospice enrollees who did not receive concurrent care, median (IQR) hospice LOS was 4 (6) days for Medicare, 5 (12) days for VA community care, and 1 (9) day for VA inpatient. The average (SD) number of weekly dialysis sessions before hospice initiation was 3.5 (2.8) compared with 2.3 (1.0) after hospice initiation.

### Hospice Diagnosis and Setting Among Medicare Users

Among all cohort patients who received Medicare-financed hospice services, 36.8% had a primary hospice diagnosis of ESKD and 63.1% had a non-ESKD diagnosis (eg, cancer). Almost half (46.8%) of cohort patients who did not receive concurrent dialysis had a primary hospice diagnosis of ESKD compared with only 6.3% of those who did receive concurrent dialysis. The VA was the predominant payer for dialysis treatments that occurred after hospice initiation, not only among those with a primary hospice diagnosis of ESKD (78.0% VA community care; 7.8% VA; 14.1% Medicare) but also among those with a non-ESKD primary hospice diagnosis (79.5% VA community care; 6.5% VA; 14.0% Medicare; eTable 1 in the Supplement).

As shown in eTable 2 in the Supplement, among all cohort patients who received Medicare-financed hospice, 86.9% received home hospice and 13.1% received inpatient hospice. The adjusted proportion who received concurrent care was 26.2% (95% CI, 25.5%-26.9%) for those in home hospice and 14.6% (95% CI, 12.9%-16.4%) for those in inpatient hospice, and the median number of concurrent hospice days was 50 and 28 days, respectively.

## Discussion

Among the 26% of members of a national veteran decedent cohort with ESKD who received hospice services, 28% received at least 1 dialysis treatment after hospice initiation. Among the 11% of cohort members who received VA-financed hospice services, rates of concurrent care were substantially higher than among the large majority of cohort members who received Medicare-financed hospice services, even after accounting for differences among veterans in these groups. Regardless of hospice payer, VA paid nearly all dialysis treatments after hospice initiation among those who received concurrent care, even among Medicare hospice beneficiaries with a non-ESKD primary hospice diagnosis. Patients who received concurrent care had a median hospice LOS of 43 days vs 4 days for those who did not. These findings highlight the VA’s important role in supporting concurrent dialysis for veterans with ESKD and suggest that Medicare’s restrictive hospice policy substantially limits access to concurrent care for beneficiaries with ESKD, even among veterans.

The findings add to the literature documenting less frequent and less timely use of hospice services among patients with ESKD compared with patients with other serious advanced illnesses, including cancer and congestive heart failure.^13,14,15,16,17,18,19,20^ Less frequent and timely use of hospice services is concerning because past research among other patient populations suggests that hospice initiation and longer hospice stays are associated with higher quality end-of-life care and more favorable bereavement outcomes.^21,22,23,24^ Several prior studies that have examined concurrent care among veterans with advanced cancer receiving cancer-focused therapies suggest that this approach may help to improve the quality of end-of-life care.^10,11^ With the exception of 1 study published more than 15 years ago,^14^ to our knowledge, this is the first study to quantify the proportion of hospice patients with ESKD receiving concurrent hospice and dialysis services and to compare hospice LOS between those who did and did not receive concurrent dialysis. Furthermore, it is the first to compare concurrent care use among hospice payers that have differing eligibility and payment policy.

Among members of this study cohort, not only were rates of concurrent care substantially higher among veterans receiving VA-financed as compared with Medicare-financed hospice services, but VA was the predominant payer for concurrent dialysis for veterans regardless of hospice payer or Medicare primary hospice diagnosis. Collectively, these findings signal both the important role of VA in supporting the delivery of concurrent care to veterans with ESKD and a substantial unmet need for concurrent care among cohort members receiving Medicare hospice services. Despite higher rates of concurrent care among cohort members receiving VA-financed hospice services, our prior qualitative analyses^25^ of hospice mentions in the VA-wide electronic health records suggest that Medicare hospice eligibility criteria and payment policy still hold considerable sway in decision-making regarding the use of hospice for patients receiving dialysis. We found that VA clinicians were often uncertain as to whether patients were eligible to continue dialysis after hospice initiation, and the real or perceived need to stop dialysis was, in some cases, a source of distress and barrier to hospice entry. Our analysis of rates of concurrent care among veterans receiving Medicare vs VA-financed hospice services, documents reliance on the VA to pay for the concurrent dialysis, regardless of primary hospice diagnosis. We suspect that there may be an even greater unmet need for concurrent dialysis among all Medicare beneficiaries, most of whom, unlike veterans, lack access to other sources of payment for hospice and/or dialysis services.

Our findings among the veteran population highlight the importance of several ongoing Center for Medicare & Medicaid Innovation (CMMI) efforts to explore the feasibility of offering concurrent hospice and dialysis services under the Medicare program. In 2020, CMMI created the Kidney Care Choices Model (KCCM), a value-based payment model in which dialysis facilities, nephrologists, and other Medicare clinicians can partner to create Kidney Contracting Entities (KCEs) to provide care in an accountable care organization framework.^26^ The KCCM, which began to enroll patients with stage 4 or 5 chronic kidney disease in January 2022, includes a *concurrent care waiver *that enables (but does not require) KCEs to waive the requirement that beneficiaries who elect the Medicare Hospice Benefit forego their right to receive Medicare-financed curative care, most notably dialysis. All expenditures incurred by Medicare (including those for dialysis treatments) are included as part of the Total Cost of Care, a major performance measure tied to the financial compensation that KCEs receive from Medicare. In the KCCM, utilization, spending, and quality measures are evaluated by CMMI will evaluate. If some KCEs elect to offer concurrent care, the initiative could offer a unique opportunity to examine the quality and cost of concurrent care among Medicare beneficiaries and to understand how its provision shapes the end-of-life experience and care of patients with ESKD and their families.

### Limitations

Our study has several limitations. First, patients were not randomized to receive Medicare- or VA-financed hospice services, and the study’s observational design cannot support causal inferences on whether liberalizing Medicare hospice eligibility requirements and payment policies would increase rates of concurrent care. Second, the proportion of patients in VA-financed hospice was quite small relative to the proportion in Medicare-financed hospice, and the groups differed in some observable ways (eg, Medicare-financed hospice patients were older). Although we were able to adjust for potential confounders that were observable (eg, age), the analyses could not account for potentially important unobserved confounders not available in source data, including social determinants of health such as caregiving and social support, detailed clinical context, and the treatment preferences of patients and families. All of these nonpolicy factors likely affect decision-making about hospice use and the timing of initiation relative to dialysis discontinuation. Third, to our knowledge, this study is the first to document rates of concurrent care among patients with ESKD, however, data comparing the quality of care for seriously ill patients with ESKD who receive concurrent hospice and dialysis vs hospice alone vs no hospice are not currently available. Fourth, our study uses a mortality follow-back design. Despite its inherent limitations,^27^ there is strong precedent for using the follow-back design to describe patterns of end-of-life care given the challenges of prospectively identifying patients most likely to die within a certain time frame.^28^ Finally, the generalizability of these study findings to non-Veteran Medicare beneficiaries with ESKD or to those with other forms of health care coverage is uncertain. Nonetheless, the high prevalence of dual VA−Medicare use among veterans with ESKD means that these study findings have broader implications for the provision of concurrent care for Medicare beneficiaries with ESKD.

## Conclusions

The findings of this retrospective cross-sectional study found that approximately 1 in 4 members of a national cohort of veterans with ESKD who received hospice services received at least 1 dialysis session after hospice initiation. Rates of concurrent care among the small minority of cohort members whose hospice services were financed by VA were substantially higher than those among cohort members receiving Medicare-financed hospice services. Furthermore, the VA was the primary payer for concurrent dialysis treatments, regardless of primary hospice payer and/or Medicare primary hospice diagnosis. Cohort members who received concurrent care had a hospice LOS of more than 1 month as compared with only 4 days for those who did not. Our findings among veterans with ESKD suggest that there is probably a substantial unmet need for concurrent care among the large majority of veterans with ESKD receiving hospice services through Medicare.

## Notes

Supplement. **eTable 1.** Concurrent Dialysis Payer by Primary Hospice Diagnosis Among Medicare-Financed Concurrent Hospice Users **eTable 2.** Concurrent Hospice Utilization and Hospice Length of Stay Among VA-Enrolled Veterans Who Had Received Maintenance Dialysis Who Received Medicare-Financed Hospice by Setting of Hospice Care **eFigure.** Unadjusted Proportions of VA-Enrolled Hospice Users Receiving Concurrent Care by Hospice Payer, Overall and Stratified by Dominant Dialysis Payer
