# Errors in Results

**DOI:** 10.1001/jamahealthforum.2022.4512

**Published:** 2022-11-11

In the Original Investigation titled “Association of Hospice Payer With Concurrent Receipt of Hospice and Dialysis Among US Veterans With End-stage Kidney Disease: a Retrospective Analysis of a National Cohort,”^1^ published online on October 21, 2022, the adjusted proportions of those receiving concurrent care in the Results were imprecise. This article was corrected online.
