# Supplementary material for: Association of Hospice Payer With Concurrent Receipt of Hospice and Dialysis Among US Veterans With End-stage Kidney Disease: A Retrospective Analysis of a National Cohort
Source: JAMA Health Forum. 2022 Oct 21;3(10):e223708. doi: 10.1001/jamahealthforum.2022.3708 (PMC9587478; doi:10.1001/jamahealthforum.2022.3708)
Supplement: Supplement. — eTable 1. Concurrent Dialysis Payer by Primary Hospice Diagnosis Among Medicare-Financed Concurrent Hospice Users eTable 2. Concurrent Hospice Utilization and Hospice Length of Stay Among VA-Enrolled Veterans Who Had Received Maintenance Dialysis Who Received Medicare-Financed Hospice by Setting of Hospice Care eFigure. Unadjusted Proportions of VA-Enrolled Hospice Users Receiving Concurrent Care by Hospice Payer, Overall and Stratified by Dominant Dialysis Payer [file jamahealthforum-e223708-s001.pdf]

Supplemental Online Content

Wachterman MW, Corneau EE, O'Hare AM, Keating NL, Mor V. Association of hospice payer with concurrent receipt of hospice and dialysis among US veterans with end-stage kidney disease: a retrospective analysis of a national cohort. *JAMA Health Forum.* 2022;3(10):e223708. doi:10.1001/jamahealthforum.2022.3708

eTable 1. Concurrent Dialysis Payer by Primary Hospice Diagnosis Among Medicare-Financed Concurrent Hospice Users

eTable 2. Concurrent Hospice Utilization and Hospice Length of Stay Among VA-Enrolled Veterans Who Had Received Maintenance Dialysis Who Received Medicare-Financed Hospice by Setting of Hospice Care

eFigure. Unadjusted Proportions of VA-Enrolled Hospice Users Receiving Concurrent Care by Hospice Payer, Overall and Stratified by Dominant Dialysis Payer

This supplemental material has been provided by the authors to give readers additional information about their work.

© 2022 Wachterman MW et al. *JAMA Health Forum.*

eTable 1. Concurrent Dialysis Payer by Primary Hospice Diagnosis Among Medicare-Financed Concurrent Hospice Users

	All (N=4050)	ESKD[a] Primary Diagnosis (N=255, 6.3%)	Non-ESKD[a] primary diagnosis (N=3795, 93.7%)
Concurrent dialysis payer			
Medicare-Financed	567 (14.0)	36 (14.1)	531 (14.0)
VA-Community Financed	3215 (79.4)	199 (78.0)	3016 (79.5)
VA-Financed	268 (6.6)	20 (7.8)	248 (6.5)

[a] End-stage kidney disease

© 2022 Wachterman MW et al. *JAMA Health Forum.*

eTable 2. Concurrent Hospice Utilization and Hospice Length of Stay Among VA[a]-Enrolled Veterans Who Had Received Maintenance Dialysis Who Received Medicare-Financed Hospice by Setting of Hospice Care

**e2a. Concurrent Hospice Use by Setting of Medicare-Financed Hospice Care (N=16465)**			
	Total Medicare-Financed Hospice (N=16465)	Medicare-Financed Home Hospice (N=14312, 86.9%)	Medicare-Financed Inpatient Hospice (N=2153, 13.1%)
Concurrent care use			
Unadjusted, % (N)	4050 (24.6)	3755 (26.2)	295 (13.7)
Adjusted,[b] % (95% CI)	24.9 (24.3, 25.6)	26.2 (25.5, 26.9)	14.6 (12.9, 16.4)
**e2b. Hospice Length of Stay by Setting of Medicare-Financed Hospice Care (N=16465)**			
Hospice length of stay, median (IQR)			
Overall, days	5 (15)	6 (17)	4 (7)
Non-concurrent hospice, days	4 (6)	4 (6)	4 (5)
Concurrent hospice, Days	47 (147)	50 (156)	28 (59)

[a] VA, Veterans Health Administration

[b] Adjusted for age, sex, race, rurality, proximity to nearest VA Medical Center, and VISN fixed effects

[c] P<0.001 for comparison versus Medicare-financed hospice

© 2022 Wachterman MW et al. *JAMA Health Forum.*

eFigure. Unadjusted Proportions of VA[a]-Enrolled Hospice Users Receiving Concurrent Care by Hospice Payer, Overall and Stratified by Dominant Dialysis Payer[b]

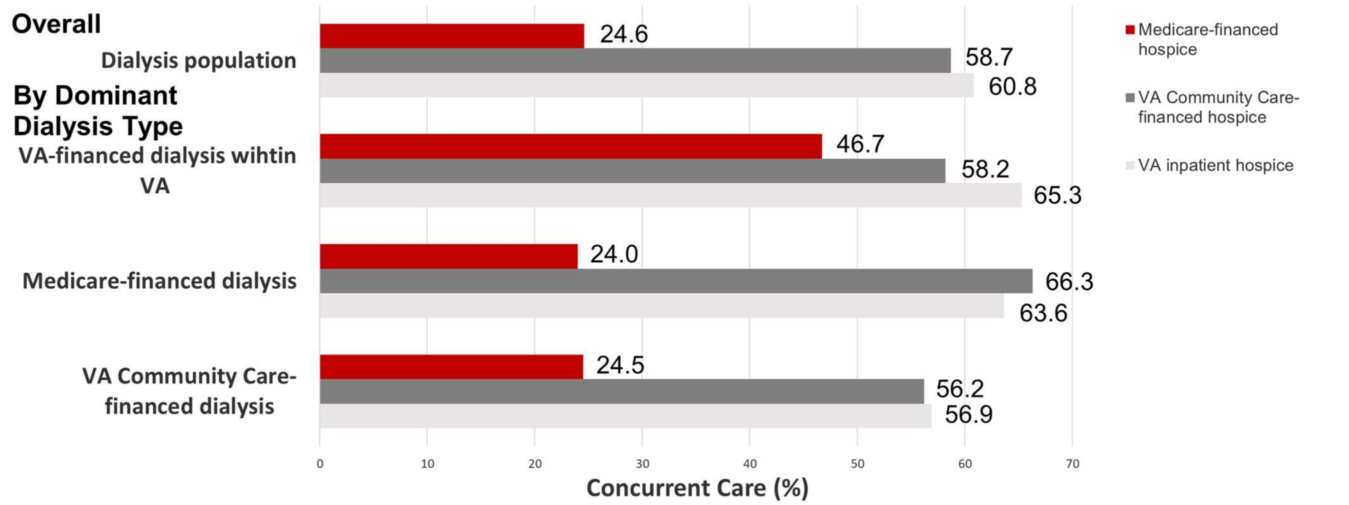

Notes: VA dialysis = dialysis in a VA dialysis facility; Medicare-financed dialysis = dialysis in a non-VA dialysis facility paid for by Medicare; VA Community Care-financed dialysis = dialysis in a non-VA dialysis facility paid for by the VA; Medicare hospice = community-based hospice services paid for by Medicare; VA Community Care-financed hospice = community-based hospice services paid for by the VA; VA inpatient hospice = hospice services in a VA inpatient facility.

[a] Veterans Health Administration; [b] Payer that financed the greatest number of dialysis treatments

© 2022 Wachterman MW et al. *JAMA Health Forum.*
